# The Inconsistencies of Germ Theories

**Published:** 1886-01

**Authors:** F. R. Campbell


### Translations.

### The Inconsistencies of Germ Theories.

Translated by F. R. Campbell, M. D.

Prof. Peter, of the Faculty of Medicine, is now giving a thorough course of lectures on a very interesting and much-disputed subject. In a remarkable opening lecture, the learned professor attempts to show the results and inconsistencies of the germ theories in general, and of those applied to special diathetic affections.

"The discovery of the *bacillus tuberculosis* is a conquest for pathological anatomy and semeiology, since the presence of the bacillus enables us to distinguish the expectoration of tuberculosis from that of common chronic bronchitis. But the enthusiasm induced by this discovery has led men far astray; they have believed that the recognition of this little rod, this supposed parasite, was about to open up a new era in the therapeutics of tubercular affections. It is a strange mistake to attempt to deduce therapeutics from pathological anatomy, which is merely the natural history of lesions, as semeiology is the clinical history of symptoms. They confuse the lesion, which is a product, with the disease, which is a process. It is like confounding medicine and surgery. The lesion is an accomplished fact; the disease is a morbid process. The surgeon treats a lesion when he reduces a fracture; the physician attacks a disease when he treats a pneumonia or a pleurisy. From a medical point of view, nothing has been added to our knowledge of therapeutics by the discovery of the bacillus. They have committed a ridiculous error in supposing that the bacillus was the cause of the disease, and that, by destroying its life, a cure might be effected."

Speaking next of the work of Cornil Malassez, Vignal, Alvarez, Lustgarten and Leyden, relating to the study of the microbes of tuberculosis, leprosy and syphilis, the professor concludes that the contagious element does not reside in the bacillus, but in the substance which surrounds it. We should not speak of the bacillus of tuberculosis, leprosy or syphilis, but should simply mention that the bacillus comes from a tubercular, leprous or syphilitic patient. The bacillus can infect us if it comes from an infected body; it is not in itself infectious. The same observations apply to cholera and rabies. The germ theory has overlooked the part which liquids play in infection. This error is strikingly illustrated in the recent investigation of cholera and rabies. The four students sent by Pasteur to Alexandria had but one object in view—to find the microbe of cholera. Who does not know the illusions and mistakes of the French savants? They thought they had found specific microbes, but these proved to be only the fibrin of the blood, as was demonstrated to them by Koch, who, more fortunate than his confreres, discovered, first in Alexandria and then in Calcutta, his second specific germ, the comma bacillus. By conclusive arguments, the French pathologists proved to Koch that the comma bacillus did not exist in cases of rapidly fatal cholera.

In these cases, two or three comma bacilli may perchance be found; they are then led to the absurd conclusion that the fewer the bacilli the more severe the disease. Would it not be more logical to suppose that, when death comes on so rapidly, the bacillus had not had time to appear? In order to escape this conclusion, Koch has called to his aid the secretion, by his bacillus, of a supposed ptomaine. But granting that it *is* the ptomaine secreted by the bacillus which causes the symptoms, the quantity of ptomaine should correspond with the number of bacilli. In every case when forced to the wall, germ theories have required for their support a humoral pathology.

And see what is being done in regard to rabies! They searched for its microbe in the saliva, and found there a dozen germs, but not the true one. They next looked for it in the medulla oblongata, and failed again. But they did discover molecular granulations. Pasteur has spoken of granulations, infinitessimally small, scattered over the medulla of a rabid animal. What a wonderful discovery! Gluge saw them twenty-five years ago, and called them *granular bodies.* They are found in all cases of myelitis during the congestive period.

Prof. Peter finished this remarkable lecture by signaling his intention to wage a merciless war against germ theories, "which have spread like parasites, which mistake the effect for the cause, a morbid product for the source of the disease, a similar for an identical."

The effects of these doctrines upon therapeutics are already quite manifest. Everybody knows the sad termination of the method of Ferrand, who experimented with the cholera in Spain, as some have done in laboratories.

As to tuberculosis, this theory compels us to consider every consumptive dangerous; to institute against him rigorous quarantines; to dose him with *germicides*, such as carbolic acid, creosote, boric acid, etc., drugs which may be *homocides* as well.

As to the social consequences, these have been seen in the recent epidemics in Italy and the south of France, where scenes of barbarism befitting another age were enacted. In Italy, it appears, proprietors demand that persons suspected of having phthisis shall vacate their property.

We must congratulate Prof. Peter upon the courageous and scholarly manner in which he defends true clinical medicine. It is those who pass their lives surrounded by disease, who do most for practical medicine; and we sincerely hope that all practical physicians will appreciate the efforts of the master who combats the errors which have filled the public mind with alarm. —*Journ. de Med. de Paris*, Dec. 8, 1885.

